# The value of a neural network based on multi-scale feature fusion to ultrasound images for the differentiation in thyroid follicular neoplasms

**DOI:** 10.1186/s12880-024-01244-1

**Published:** 2024-03-27

**Authors:** Weiwei Chen, Xuejun Ni, Cheng Qian, Lei Yang, Zheng Zhang, Mengdan Li, Fanlei Kong, Mengqin Huang, Maosheng He, Yifei Yin

**Affiliations:** 1 Department of Medical Ultrasound, Affiliated Hospital of Nantong University, 226001 Nantong, P.R. China; 2 Department of Medical Ultrasound, Affiliated Hospital of Jiangsu University, 212000 Zhenjiang, P.R. China; 3 Department of Medical Ultrasound, Affiliated Hangzhou First People’s Hospital, 310006 Hangzhou, P. R. China; 4 Shanghai Soundwise Information Technology Co., Ltd, 200000 Shanghai, P.R. China; 5 Department of Medical Ultrasound, Xuzhou Central Hospital, 221000 Xuzhou, P.R. China

**Keywords:** Follicular thyroid carcinoma, Ultrasound images

## Abstract

**Objective:**

The objective of this research was to create a deep learning network that utilizes multiscale images for the classification of follicular thyroid carcinoma (FTC) and follicular thyroid adenoma (FTA) through preoperative US.

**Methods:**

This retrospective study involved the collection of ultrasound images from 279 patients at two tertiary level hospitals. To address the issue of false positives caused by small nodules, we introduced a multi-rescale fusion network (MRF-Net). Four different deep learning models, namely MobileNet V3, ResNet50, DenseNet121 and MRF-Net, were studied based on the feature information extracted from ultrasound images. The performance of each model was evaluated using various metrics, including sensitivity, specificity, positive predictive value (PPV), negative predictive value (NPV), accuracy, F1 value, receiver operating curve (ROC), area under the curve (AUC), decision curve analysis (DCA), and confusion matrix.

**Results:**

Out of the total nodules examined, 193 were identified as FTA and 86 were confirmed as FTC. Among the deep learning models evaluated, MRF-Net exhibited the highest accuracy and area under the curve (AUC) with values of 85.3% and 84.8%, respectively. Additionally, MRF-Net demonstrated superior sensitivity and specificity compared to other models. Notably, MRF-Net achieved an impressive F1 value of 83.08%. The curve of DCA revealed that MRF-Net consistently outperformed the other models, yielding higher net benefits across various decision thresholds.

**Conclusion:**

The utilization of MRF-Net enables more precise discrimination between benign and malignant thyroid follicular tumors utilizing preoperative US.

## Introduction

In clinical practice, thyroid follicular neoplasms are categorized primarily as follicular thyroid adenoma (FTA) or follicular thyroid carcinoma (FTC). FTC, the second most prevalent type of differentiated thyroid cancer, accounts for approximately 10-20% of all thyroid cancers [1]. Patients typically exhibit distant metastasis through hematogenous dissemination, with the lungs and bones being the most commonly affected sites [2, 3]. According to the published literature, the annual incidence rate of follicular thyroid cancer in many countries, including the United States, is approximately 0.8 per 100,000 people, with a male-to-female ratio of 1:2.5 [4]. Although the incidence of FTC is lower than that of papillary thyroid carcinoma (PTC), FTC is associated with a greater rate of distant metastasis and mortality [5–7].

Currently, the main diagnostic approaches for thyroid follicular neoplasms include conventional ultrasound, fine-needle aspiration cytology (FNAC), diagnostic surgical excision, CT, and MRI. Conventional ultrasound examination offers advantages such as non-invasiveness, lack of radiation exposure, multi-angle imaging, and affordability. It is the most commonly employed imaging modality for diagnosing thyroid diseases in clinical practice, surpassing CT, MRI, and other methods. However, distinguishing between FTA and FTC based on nodule size, shape, echogenicity, margin characteristics, calcification patterns, and vascularity is challenging, as both entities exhibit significant overlap in ultrasonic images [8–11]. Moreover, FNAC, which is often limited by the site of puncture, faces challenges in providing a definitive pathological diagnosis due to the morphological similarities between FTA and FTC [12]. The differential diagnosis between these two tumors depends on determining the presence of vascular or extrathyroidal tissue invasion and lymph node or distant metastasis. Although a standardized clinical thyroid imaging data reporting system known as the thyroid imaging reporting and data system (TI-RADS) exists, the interpretation of ultrasound images still exhibits some subjectivity among different ultrasound practitioners, leading to potential misdiagnosis or missed diagnosis [13]. The guidelines established by the American Thyroid Association recommend diagnostic surgical excision as a well-established standard for managing follicular neoplasms or suspicious cases [14]. Pathology after surgical resection often reveals that, among patients initially diagnosed with follicular tumors, up to 80% have follicular adenomas. This finding indicates that a significant proportion of patients, despite having a benign condition, undergo diagnostic thyroid lobectomy. Therefore, it is crucial to preoperatively differentiate between these two conditions to avoid such overtreatment in patients with benign disease. Moreover, this distinction can reduce the misdiagnosis rate in malignant patients, ultimately improving survival rates.

In recent years, artificial intelligence has rapidly advanced as an innovative tool and has found widespread application across various medical specialties [15]. In the realm of ultrasound diagnostics, researchers can employ computer algorithms to automatically extract image features that may be imperceptible to the human eye or that are challenging for ultrasound practitioners to articulate verbally. These extracted features can be translated into reliable data, providing insights into the underlying pathophysiology and offering valuable information for disease diagnosis and prognosis [16–18]. Yadav et al. compared the performance of 64 despeckling filter algorithms for analyzing ultrasound images of thyroid nodules by calculating the structure and preserving the edge. The results showed that the fast bilateral filter and edge-preserving smoothing filters had the best performance in preserving image structures, such as the edges and margins of benign and malignant thyroid tumors [19]. While numerous studies have focused on the detection and automated diagnosis of thyroid nodules to distinguish between benign and malignant cases, there is still a paucity of research on distinguishing benign and malignant thyroid follicular tumors [20–22].

To the best of our knowledge, Shin et al. investigated the use of machine learning by employing manual nodule segmentation to screen and extract features, subsequently incorporating them into support vector machine (SVM) and artificial neural network (ANN) classifier models [23]. The results demonstrated an accuracy of 74.1% for the ANN model and 69.0% for the SVM model. Seo et al., on the other hand, employed deep learning techniques to identify ultrasound images of thyroid follicular tumors [24]. They proposed the use of convolutional neural networks (CNNs) to detect specific morphological features within the border regions of thyroid follicular tumors, leveraging image selection subsampling and datasets provided by the tumor boundaries. Alabrak et al. utilized a CNN model to extract 886 pathological images from 43 patients’ Bethesda Class IV nodules (527 FTC images, 359 FTA images) [25]. Among these, 708 images were used as the training set, and 108 images were used as the test set. The input images were color images with a resolution of 256 × 256 pixels. The convolutional and pooling layers automatically extract image features, ultimately providing a binary classification for FTA and FTC. The results demonstrated an accuracy of 78.0%, a sensitivity of 88.4%, a specificity of 64.0%, and an AUC of 0.870 in distinguishing and diagnosing FTA and FTC. Deng et al. employed four deep neural network (DNN) models (Resnet50, Densenet121, EfficientNet, and Resnext50), while Chan et al. compared three convolutional neural network (CNN) models (InceptionV3, ResNe101, and VGG19) and evaluated the diagnostic efficacy of two clinical experts with over 20 years of experience [26, 27]. The outcomes of these studies collectively indicate that CNN models can serve as auxiliary diagnostic tools for preoperative differentiation between FTA and FTC. However, both of these studies possess certain limitations that necessitate further improvement. For instance, Shin and Seo et al. manually extracted sampled images along the lesion contour, potentially overlooking certain features during the extraction process. Thyroid experts emphasize the importance of considering the lesion envelope, specifically the marginal zone of the segmented region, as a critical factor for distinguishing between FTA and FTC [28, 29]. Notably, recent advancements in deep learning have revolutionized cancer diagnosis, with multi-scale fusion neural networks gaining considerable attention due to their exceptional feature extraction capabilities [30–33].

In our study, we devised a novel neural network model called the multi-scale fusion network (MRF-Net), which incorporates the fusion of multi-scale features. The primary contribution of our research lies in integrating ultrasound findings with a neural network model that incorporates multi-scale fusion. This combination aims to increase the accuracy of preoperative diagnosis of follicular thyroid tumors. The significance of our study extends to the realm of personalized treatment for patients and the rational allocation of medical resources. By doing so, we can avoid the overtreatment of benign cases and reduce the rate of misdiagnosis of malignant cases, ultimately improving overall survival rates. Herein, we provide detailed elucidations of the notable advantages offered by this model:

- The application of multi-scale image processing methods significantly enhances the structural characteristics of images, resulting in improved accuracy and efficiency in image classification.
- The incorporation of the Rescale Enhancement Image Module (REI) enables the suppression of noise interference along with the simultaneous enhancement of boundary features. This feature facilitates the identification and differentiation of various elements within the image.

## Materials and methods

### Study population

Patient data for this retrospective study were collected from two hospitals from August 2009 to June 2022. The data consisted of patients who underwent surgical pathology for thyroid follicular tumors. The collected information included patient demographic information, 2D ultrasound images, immunohistochemical results from pathology sections, and other relevant data. Ethical approval was obtained from the institutional review board, and the requirement for informed consent was waived for the two study populations.

**Fig. 1:**
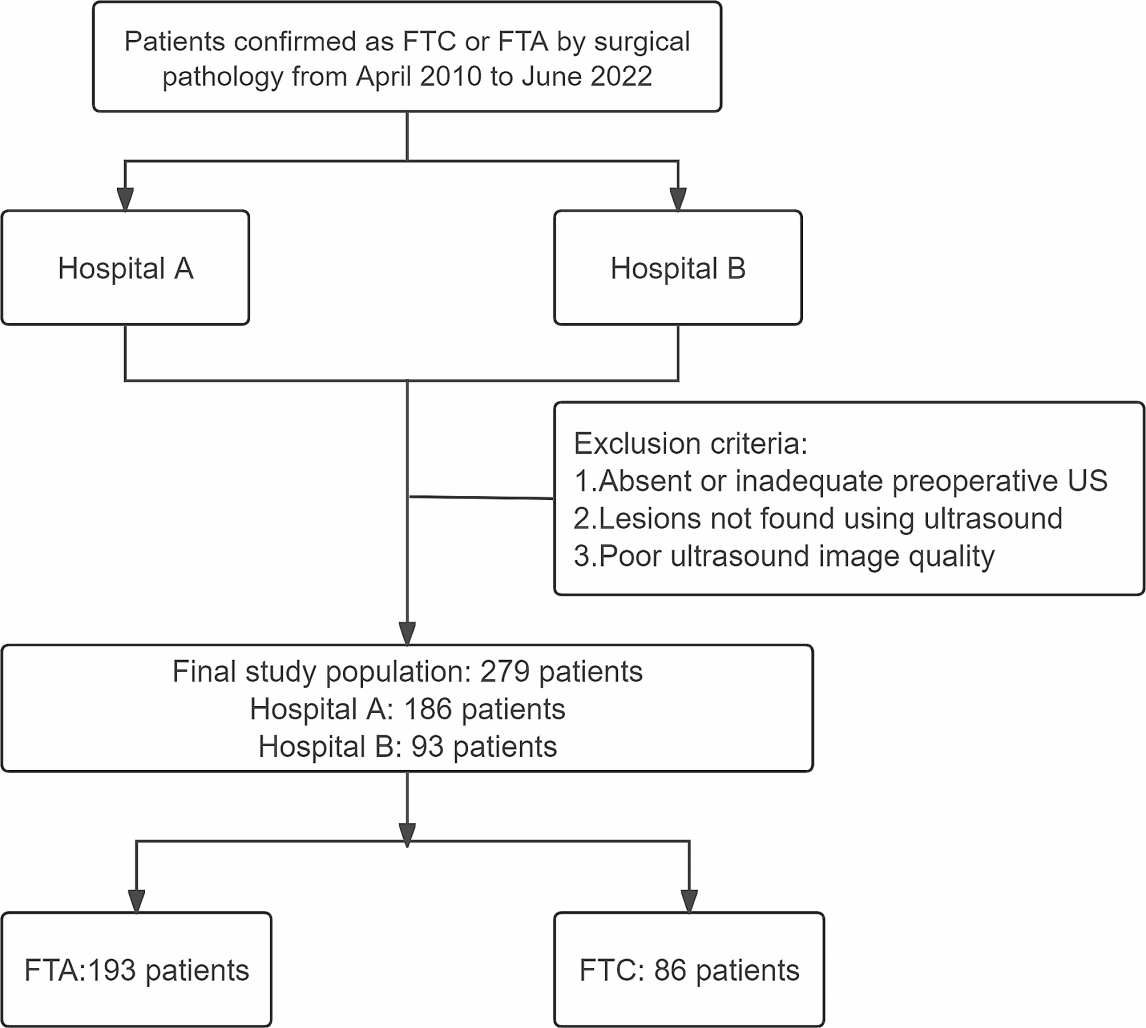
A flow chart of data collection

All patients included in the study met the following selection criteria: (1) underwent their initial surgery and received a pathological diagnosis of either FTC or FTA; (2) underwent a preoperative ultrasound examination at one of the two hospitals, and the ultrasound images obtained were of sufficient quality. Some patients were excluded from the study due to missing preoperative ultrasound image information, undetected thyroid nodules during the preoperative ultrasound examination, or inadequate image quality. Ultimately, a total of 279 patients (Fig. 1) met the eligibility criteria and were included in the study; 193 patients were diagnosed with FTA, and 86 patients were diagnosed with FTC.

### Image collection and preprocessing

**Fig. 2:**
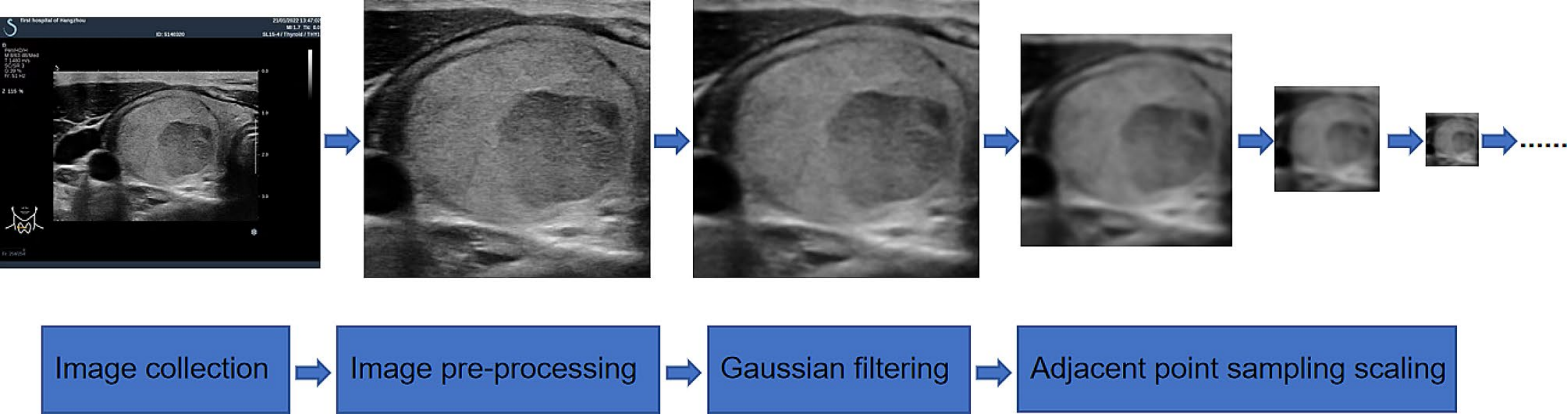
A flow chart of image processing

Experienced radiologists acquired preoperative two-dimensional images of all thyroid nodules using an ultrasound image archive workstation. Subsequently, experienced ultrasound physicians reviewed the images and retrospectively selected the maximum transverse and longitudinal plane images for each nodule. Whenever uncertainties arose regarding the images, a senior radiologist with more than 20 years of experience was consulted for further evaluation. Finally, all the collected images were cropped to remove extraneous information, ensuring that only the nodule remained at the center of the image (Fig. 2).

### The proposed multi-scaled image learning method

In this study, we propose a deep learning network that utilizes multi-scale images for the classification of FTC and FTA. First, it is crucial to review the principles of multi-scale image analysis, as it plays a significant role in our current task. Multi-scale image analysis has the ability to enhance image features effectively, leading to improved classification accuracy.

#### Design of multi-scale image processing blocks

Multi-scale image processing involves decomposing and reconstructing an original image into different scales to extract features at various scales, thereby enabling comprehensive and accurate image analysis and processing. By decomposing the original image, multi-scale image processing captures information about different scales, facilitating the representation of features such as edges and textures. Various decomposition methods can be employed, including pyramid decomposition and wavelet decomposition.

The key advantages of multi-scale image processing technology are as follows: First, it enhances the reliability and robustness of image features by capturing features and details at different scales through multi-scale processing. This contributes to improved reliability in feature extraction. Second, it enhances algorithm robustness by enabling adaptability to changes at different scales, thereby enhancing the algorithm’s generalization ability. Third, the impact of noise is reduced by decomposing the original image and eliminating high-frequency components, leading to reduced noise interference and improved algorithm accuracy. Finally, the algorithm efficiency is enhanced by allowing calculations to be performed at different scales.

To optimize the performance of our MRF-Net, we carefully selected the following hyperparameters for our CNN models. The learning rate was initially set to 0.001, with a dynamic adjustment mechanism that reduces the rate by a factor of 0.1 if the validation loss plateaus for more than 10 epochs. We used a batch size of 32 to balance the computational efficiency and model performance. The models were trained using the Adam optimizer due to its adaptability in adjusting learning rates for different parameters. The dropout rate was set to 0.5 in fully connected layers to prevent overfitting. Furthermore, we applied L2 regularization with a lambda value of 0.001 to penalize large weights in the network. The convolutional layers used ReLU (Rectified Linear Unit) activation functions for introducing non-linearity, while the final output layer utilized a softmax activation function for multi-class classification. The models were trained for a total of 200 epochs, or until no significant improvement in validation accuracy was observed.

**Fig. 3:**
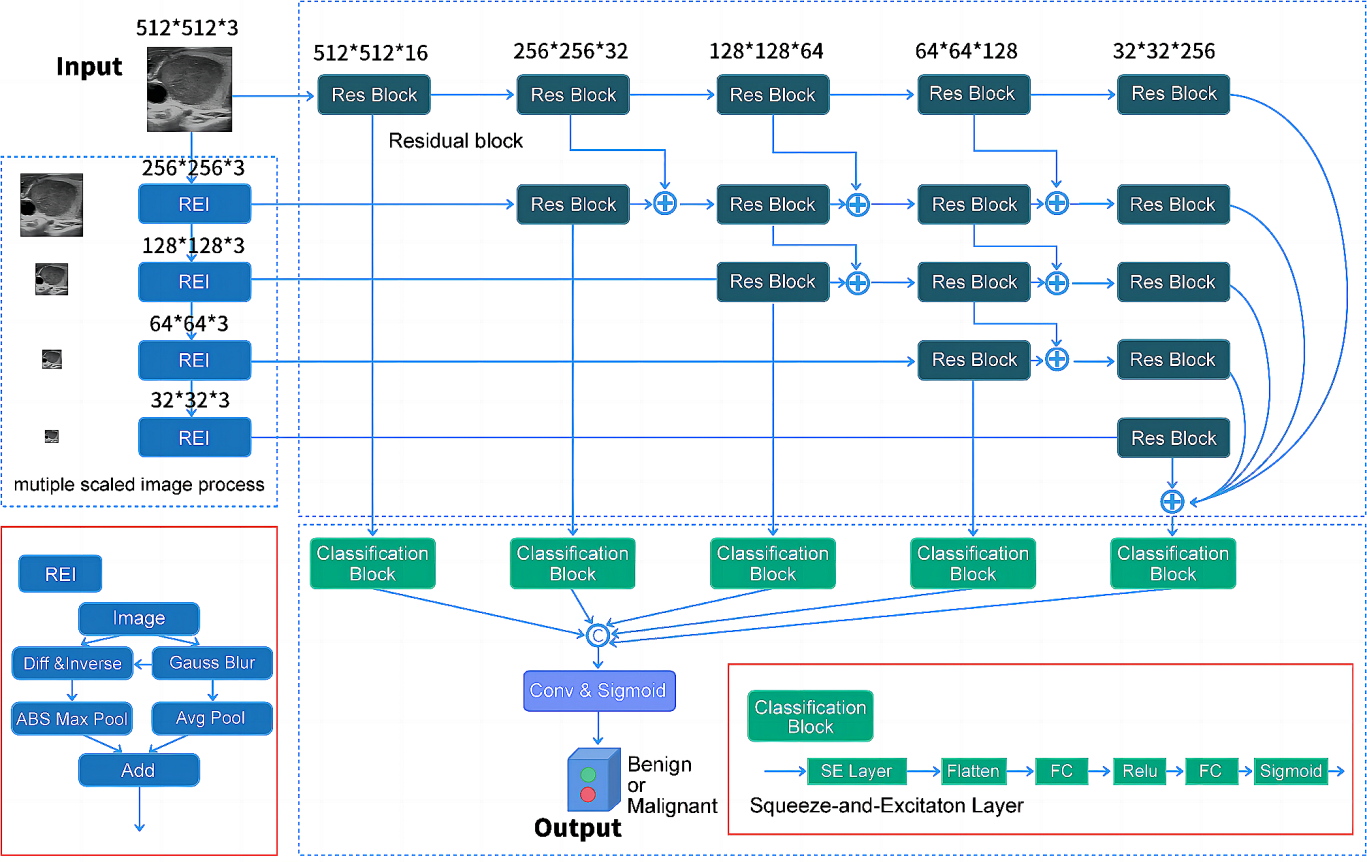
Diagram of overall workflow of model training and validation

In this paper, we propose the use of the REI to enhance the head layer of the network architecture. Utilizing a Gaussian pyramid structure, similar to that in Fig. 3, the input image undergoes multi-scale processing to improve its representation.

The specific method is as follows:

1. Gaussian filtering was applied to the image, resulting in a Gaussian distribution (Fig. 2).
2. The difference between the Gaussian image and the input image is computed, and the negative values are subsequently obtained to yield the difference images.
3. Average pooling is conducted on the gaussian image, and its dimensions are reduced by half.
4. Utilize max absolute value pooling on the difference image, reducing its dimensions by half.
5. The reduced gaussian image and the difference image are combined to generate a new image.
6. The features of the new image are enhanced through structural part enhancement.
7. The enhanced image is output as the final result.

Compared with traditional multi-scale structures, there are two merits:

1. The difference image undergoes absolute maximum pooling, which effectively preserves the essential structure while suppressing noise.
2. The difference image is subsequently added to the Gaussian image, thereby enhancing the structural features of small-scale images.

When applying the Gaussian filtering as part of our image pre-processing, we used a standard deviation (σ) value of 1.5 for the Gaussian kernel. This value was chosen to effectively balance the smoothing effect and preservation of image details, crucial for maintaining the integrity of features relevant to our classification task.

These steps are closely associated with the characteristics of the filtering structure in FTC and FTA. Some filtering structures exhibit unclear boundaries due to speckle noise interference. Applying a general Maxpool operation can cause these boundaries to vanish. Therefore, in multi-scale image processing, enhancing boundaries is highly important.

#### Multi-scale feature fusion module

The notion of multi-scale feature fusion was proposed for a considerable time and was initially implemented in the universal neural enhancement technology (UNET) segmentation network [34]. Additionally, in the realm of object detection, Kaiming He introduced the feature pyramid network (FPN) structure in RetinaNet, which exemplifies the concept of multi-scale feature fusion [35]. However, there are notable distinctions between these two approaches. The FPN is primarily employed for object detection, while the UNET is utilized for segmentation. The FPN produces multiple layers of output, whereas the UNET provides output solely in the final layer. Furthermore, their upsampling methods differ, with direct interpolation used in one case and up-convolution and optimizing parameters used in the other. While the FPN employs an addition operation for skip connections, the UNET utilizes concatenation.

Upon examining the dataset, we observed that the lesion targets in FTA and FTC tended to be relatively large, necessitating a substantial receptive field for accurate identification. As a result, reversing the fusion of high-level features lacks significance in this context. Drawing inspiration from the FPN, we adopted an “add” fusion method during the downsampling process of each layer’s output. In contrast to UNET and FPN, we omitted the upsampling step and retained the fundamental features of FTC and FTA. By feeding the outcomes of each layer into the inference, we reduce the computational burden by eliminating the upsampling module.

### Datasets and experimental setting

The experimental images were sourced from two hospitals. All the images were cropped to the minimum bounding square that encompassed the FTA and FTC lesions. Subsequently, the images were resized to 256 × 256 pixels. Of these, 283 FTA images and 122 FTC images were allocated for the training and validation sets, respectively, while 76 images were designated as the test set (Table 1). Fivefold random cross-validation was employed for training the images. The 405 training sets were randomly divided into five subsets, with one subset reserved for validation and the remaining four subsets utilized for training (Table 2). The group that yielded superior results in the validation set was ultimately selected as the final result group. During the model training phase, the batch size was set to 2, the number of epochs was set to 1000, and the learning rate was set to 1e-4. Considering that the dataset is not large, the batch size and learning rate settings are relatively small.

**Table 1:** Distribution of the dataset

	Training Set	Validation Set	Test Set
FTC(label1)	226	57	42
FTA(label0)	98	24	34
Total	324	81	76

**Table 2:** Distribution of the data for the five subsets

	Subset 1	Subset 2	Subset 3	Subset 4	Subset 5
FTC(label1)	56	57	56	57	57
FTA(label0)	25	24	25	24	24

### Evaluation methods

The performance of our model was assessed by calculating several metrics, including sensitivity, specificity, positive predictive value (PPV), negative predictive value (NPV), accuracy, and F1 score. Additionally, we utilize a confusion matrix to calculate the true positive rate and false positive rate of the model. To evaluate the overall performance of the model, we plotted an ROC curve and calculated the area under the curve (AUC) metric. The F1 score, which is the harmonic mean of precision and recall, is considered a comprehensive metric that combines both precision and recall, making it highly useful for evaluating model performance. Furthermore, we employed decision curve analysis (DCA) to assess the value of the model. By comparing the DCA curves of different models, we can determine which model is better suited for specific decision scenarios and select the optimal decision threshold [36].

$$ \mathbf{S}\mathbf{e}\mathbf{n}\mathbf{s}\mathbf{i}\mathbf{t}\mathbf{i}\mathbf{v}\mathbf{i}\mathbf{t}\mathbf{y}\left(\mathbf{r}\mathbf{e}\mathbf{c}\mathbf{a}\mathbf{l}\mathbf{l}\right) =\frac{\mathbf{T}\mathbf{P}}{\mathbf{T}\mathbf{P}+\mathbf{F}\mathbf{N}}$$

$$ \mathbf{S}\mathbf{p}\mathbf{e}\mathbf{c}\mathbf{i}\mathbf{f}\mathbf{i}\mathbf{c}\mathbf{i}\mathbf{t}\mathbf{y} =\frac{ \mathbf{T}\mathbf{N}}{\mathbf{T}\mathbf{N}+\mathbf{F}\mathbf{P}}$$

$$ \mathbf{P}\mathbf{P}\mathbf{V}\left(\mathbf{P}\mathbf{r}\mathbf{e}\mathbf{c}\mathbf{i}\mathbf{s}\mathbf{i}\mathbf{o}\mathbf{n}\right) =\frac{ \mathbf{T}\mathbf{P}}{\mathbf{T}\mathbf{P}+\mathbf{F}\mathbf{P}}$$

$$ \mathbf{N}\mathbf{P}\mathbf{V}=\frac{\mathbf{T}\mathbf{N}}{\mathbf{T}\mathbf{N}+\mathbf{F}\mathbf{N}}$$

$$ \mathbf{A}\mathbf{c}\mathbf{c}\mathbf{u}\mathbf{r}\mathbf{a}\mathbf{c}\mathbf{y}=\frac{\mathbf{T}\mathbf{P}+\mathbf{T}\mathbf{N}}{\mathbf{T}\mathbf{P}+\mathbf{T}\mathbf{N}+\mathbf{F}\mathbf{P}+\mathbf{F}\mathbf{N}}$$

$$ \mathbf{F}1=\frac{ 2 \ast \mathbf{P}\mathbf{P}\mathbf{V} \ast \mathbf{S}\mathbf{e}\mathbf{n}\mathbf{s}\mathbf{i}\mathbf{t}\mathbf{i}\mathbf{v}\mathbf{i}\mathbf{t}\mathbf{y}}{\mathbf{P}\mathbf{P}\mathbf{V} + \mathbf{S}\mathbf{e}\mathbf{n}\mathbf{s}\mathbf{i}\mathbf{t}\mathbf{i}\mathbf{v}\mathbf{i}\mathbf{t}\mathbf{y}}$$

In our evaluation, TP (true positive) corresponds to the number of accurately classified FTC cases, while TN (true negative) refers to the number of accurately classified FTA cases. On the other hand, FP (false positive) and FN (false negative) indicate the number of incorrectly classified FTC/FTA cases.

Based on the aforementioned information, sensitivity (also known as recall) represents the model’s ability to accurately predict FTC samples out of all samples that truly belong to the FTC category. On the other hand, specificity denotes the model’s ability to correctly identify FTA samples out of all samples that genuinely fall into the FTA category. The precision (also known as the PPV) corresponds to the proportion of samples correctly classified as FTC by the model out of all samples predicted as FTC, while the NPV indicates the proportion of samples accurately identified as FTA among all samples predicted as FTA by the model. The accuracy represents the overall proportion of samples correctly predicted by the model out of the entire sample set, and the F1 value serves as an evaluation metric that considers both precision and recall simultaneously.

### Statistical analysis

Statistical analysis was performed using SPSS 22 software (SPSS, 1989; Apache Software Foundation, Chicago, IL, USA). The Delong test was also conducted to assess any significant differences in diagnostic performance among the various models. A two-sided p-value of less than 0.05 was considered to indicate statistical significance. ROC curves were generated to determine the area under the ROC curve (AUROC), cutoff values, sensitivity, specificity, positive predictive value (PPV), and negative predictive value (NPV).

## Results

**Fig. 4:**
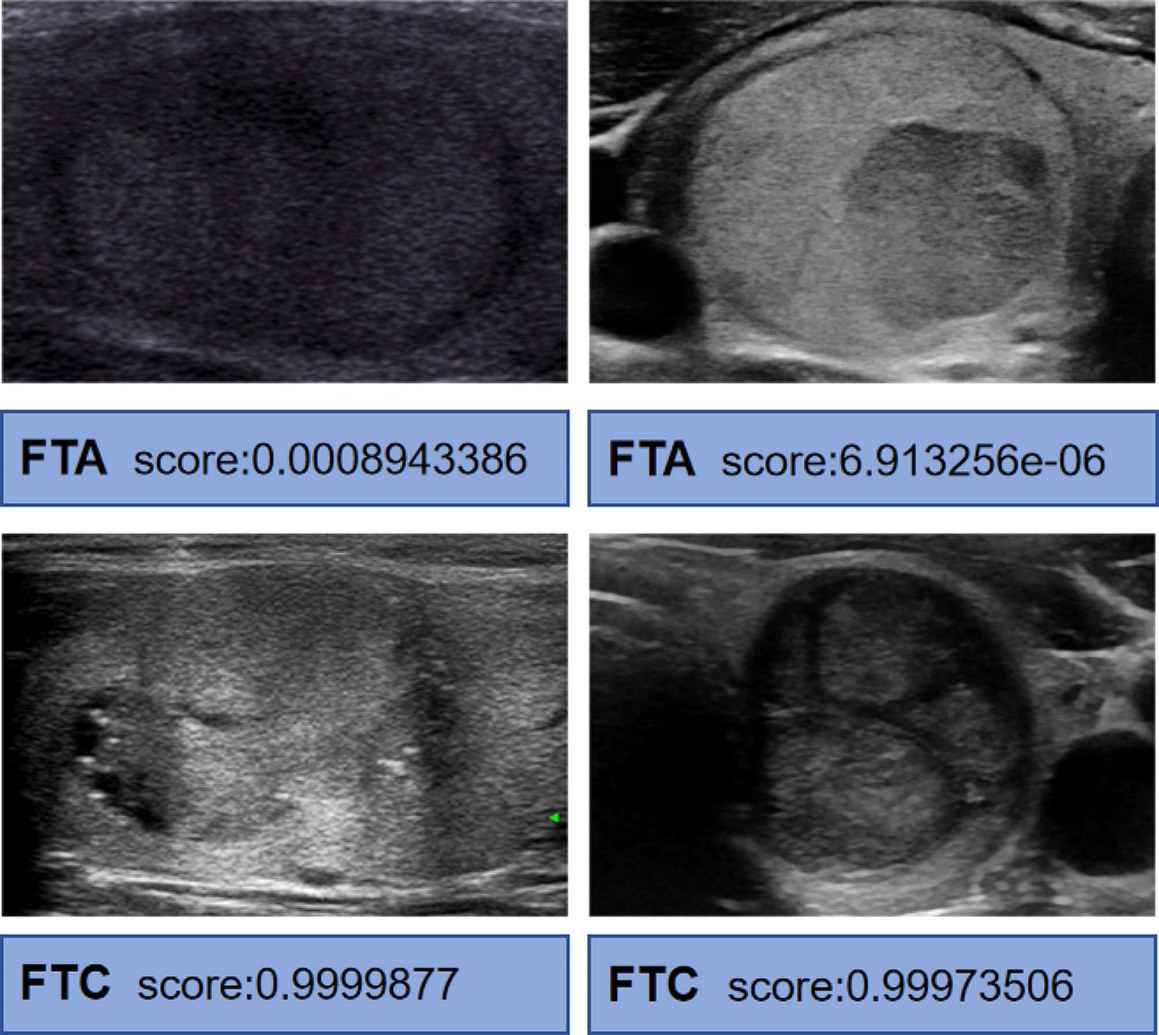
Examples of the case tested

Two criteria were used to evaluate the score of the model: 0.5 was used as the cutoff for identification, a deviation in the score to 0 was considered benign, and a deviation in the score to 1 was considered malignant. Figure 4 shows the ultrasound image cases and model scores of several tests. The results showed that our model performed well and could accurately distinguish between FTC and FTA in two similar ultrasound images. Based on the experimental data provided, MRF-Net outperforms other commonly used neural network models, exhibiting the highest sensitivity (79.41%), specificity (90.24%), PPV (87.1%), and NPV (84.1%) (Table 3). The accuracy, a fundamental metric for evaluating predictive performance, was reported as 85.3% for MRF-Net, surpassing the other models presented in Table 3. This indicates that the MRF-Net model is more likely to correctly predict outcomes than are the other models. To account for potential class imbalances within the dataset, the F1 value was calculated, providing a comprehensive evaluation of the models’ performance. MRF-Net achieved an impressive F1 value of 83.08%, suggesting a well-balanced prediction of both FTA and FTC outcomes, making it a reliable predictor for the dataset.

87.1

**Table 3:** Comparison of experimental results

(%)	MobileNetV3	Resnet50	DenseNet121	MRF-Net
Sensitivity	73.53	76.47	76.47	**79.41**
Specificity	70.73	75.61	80.49	**90.24**
PPV	67.57	72.22	76.47	87.1
NPV	76.32	79.49	80.49	**84.1**
Accuracy	72	76	78.67	**85.3**
F1 value	70.42	74.29	76.47	**83.08**

**Fig. 5:**
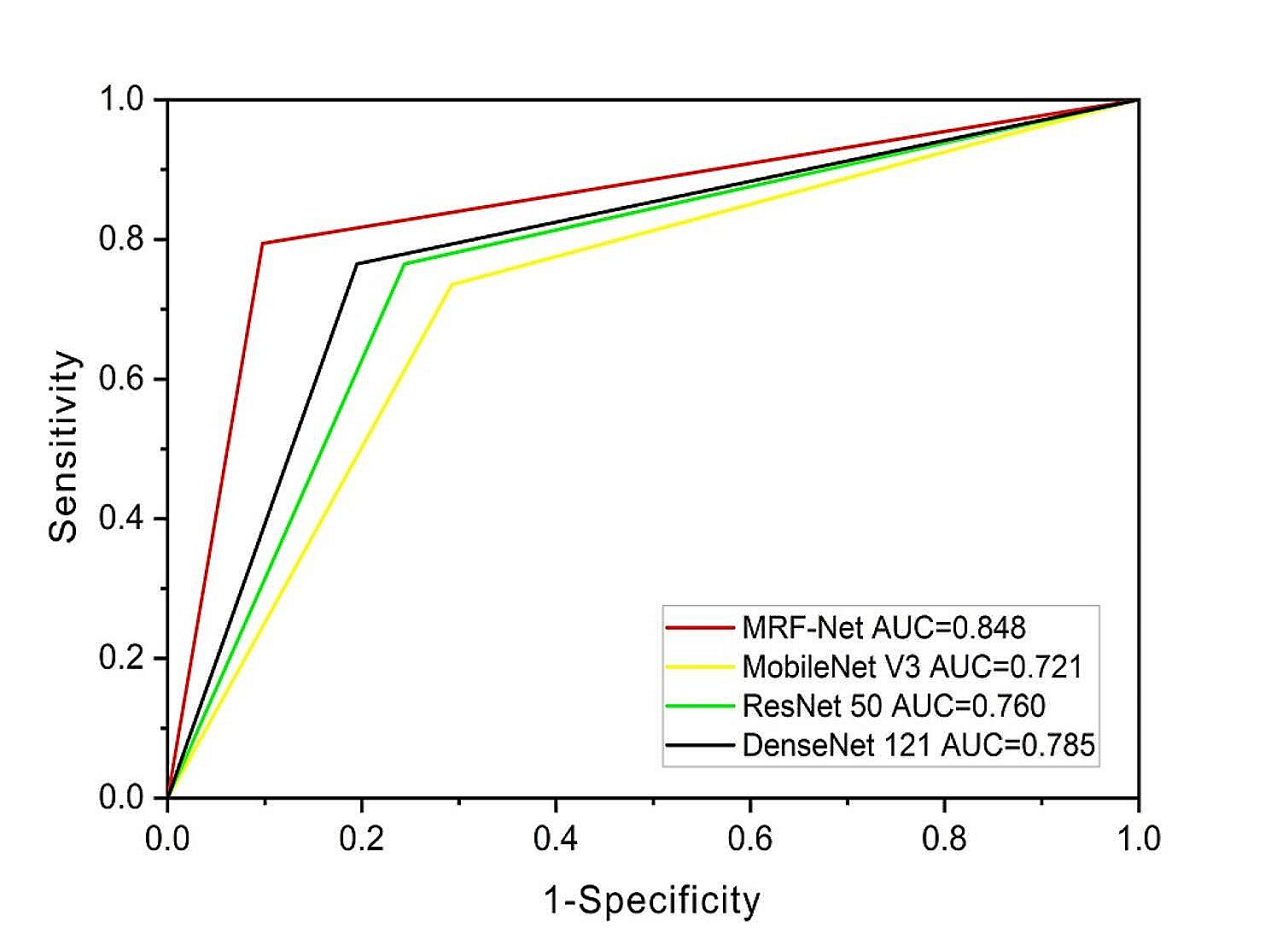
The AUC values of different models

**Fig. 6:**
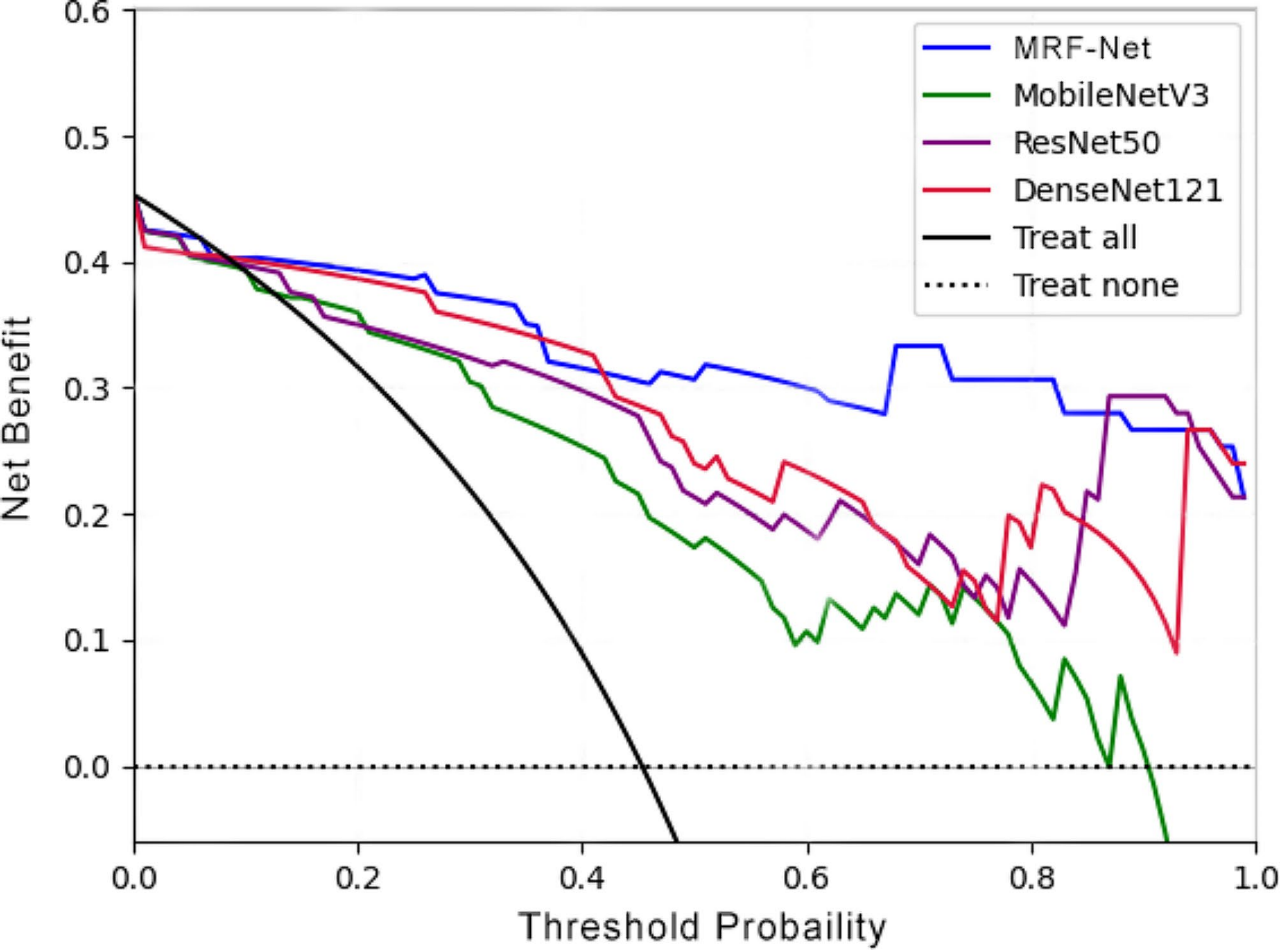
The DCA curves of different models

**Fig. 7:**
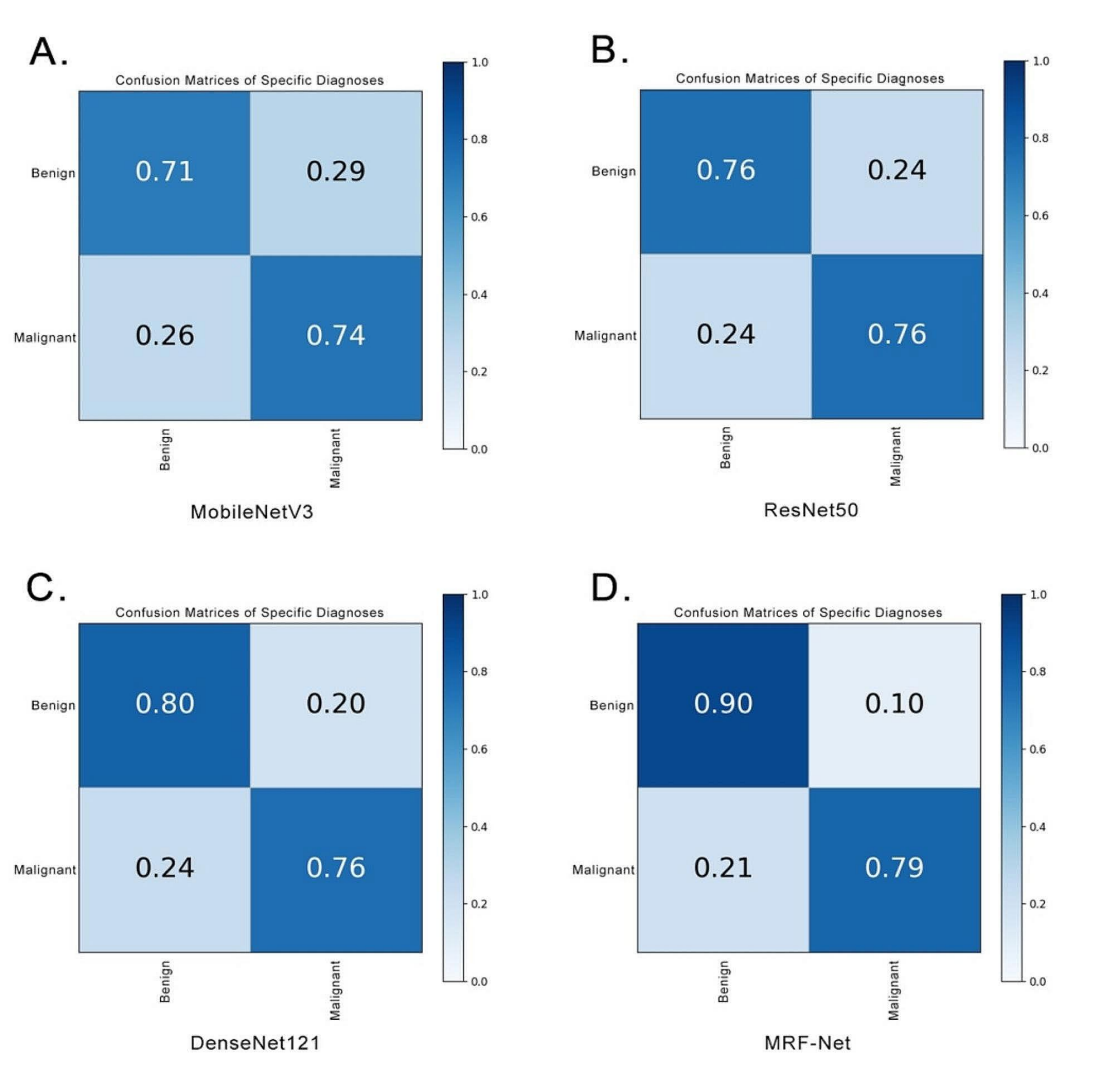
The confusion matrix charts for MobileNetV3 (**A**), Resnet50 (**B**), DenseNet121 (**C**) and MRF-Net (**D**)

Figure 5 displays the experimental results of MobileNet V3, ResNet50, and DenseNet121, with their ROC curves closely clustered and an overall AUC ranging from 0.721 to 0.785. Among these models, MRF-Net (red line) exhibited relatively better performance, with an overall AUC of 0.848, the highest among them. A comparison of the ROC curves revealed that the AUC of MRF-Net was significantly greater than that of MobileNet V3 and ResNet50, with corresponding p values of 0.0007 and 0.0054, respectively. Moreover, DCA curve analysis demonstrated that the net benefit curve of MRF-Net consistently outperformed that of the other models across different decision thresholds (Fig. 6). These findings indicate that MRF-Net is a superior classification model, particularly for distinguishing between benign and malignant thyroid follicular tumors. This finding suggested that MRF-Net would provide greater benefits to patients and offer valuable support to doctors in making informed decisions in such cases. Furthermore, the performance of the four models was assessed by analyzing their confusion matrices. As depicted in Fig. 7, the horizontal axis represents the model’s output results, with dark blue squares indicating the probability of correct predictions for FTA or FTC. Notably, MRF-Net exhibited higher true positive and true negative rates than did the other three models, while its false positive and false negative rates were correspondingly lower.

The aforementioned findings indicate that our neural network exhibits a high level of accuracy and reliability in differentiating between benign and malignant thyroid follicular tumors. Notably, the utilization of multi-scale feature fusion in our network design further enhances its performance in this task. This research introduces a novel approach for discriminating between benign and malignant thyroid nodules in clinical medicine, offering promising prospects for practical applications.

## Discussion

Accurately distinguishing between benign and malignant thyroid follicular tumors before surgery is crucial for clinical treatment and subsequent therapy selection. If the model-assisted diagnosis suggests a greater possibility of FTA, patients and clinical physicians may choose conservative drug treatment or partial thyroidectomy to reduce unnecessary harm and improve quality of life. Despite the use of preoperative ultrasonography and fine needle aspiration cytology, the effectiveness of these methods in differentiating thyroid FTC from FTA has been limited. According to the investigation results of some researchers, it is difficult to identify FTA and FTC even in pathology and other imaging examinations, and their research results show low accuracy. These tumors exhibit visual similarities on ultrasound images, posing challenges even for experienced ultrasonographers. Moreover, the diagnostic accuracy of these methods is constrained by the restricted range of puncture positions.

Currently, there is limited research on follicular thyroid tumors, and further exploration is needed. Previous studies have used machine learning to manually outline lesions and analyze the benign or malignant nature of follicular thyroid tumors, achieving an accuracy rate of 74.1%. However, this approach may result in the loss of edge image feature extraction, thereby reducing the accuracy. To address these limitations, our study proposes a novel deep learning network based on multi-scale images for FTC and FTA classification. The lesion was placed in the middle of the cropping box, and the integrity of the edge image features was preserved. In contrast to conventional methods, we enhance the head layer of the network architecture by incorporating a stretch-enhanced image (REI) module. Our approach involves employing a Gaussian pyramid-like structure to process the input image at multiple scales. We perform absolute maximum pooling on the differential image to preserve key structures, suppress noise, and reintegrate it with the Gaussian image to enhance the structural features of small-scale images. The results demonstrated a high level of sensitivity (79.41%) and specificity (90.24%) achieved by our model, underscoring its potential for clinical application. Moreover, we also compared MRF-Net with the latest model Vision Transformer, and the results similarly showed that the former showed better results. By combining ultrasound and artificial intelligence data to construct a diagnostic prediction model based on clinical and imaging data, establishing an FTC/FTA diagnostic system is a key focus in future research on follicular tumors. However, the quantity and quality of images are crucial for artificial intelligence models, necessitating collaboration to increase multi-center database resources. Improving the accuracy of preoperative diagnosis of follicular thyroid tumors is highly important for personalized patient treatment and the rational allocation of medical resources.

Nevertheless, our study has several limitations. First, the sample size was relatively small, consisting of 279 patients’ ultrasound images, with 324 images in the training set, 81 images in the validation set, and 76 images in the test set. Therefore, expanding the dataset is necessary to enhance the generalizability of the study. Second, as a retrospective study, selection bias cannot be entirely eliminated. Finally, the ultrasound images were obtained from two hospitals, potentially leading to variations in image quality due to differences in machine models and physician expertise, which could impact the data results.

## Conclusion

The neural network incorporating multi-scale integration exhibited outstanding performance in distinguishing FTC from FTA, providing valuable support for clinical diagnosis and treatment decision-making.

## Data Availability

No datasets were generated or analysed during the current study.

## Abbreviations

FTA: follicular thyroid adenoma
FTC: follicular thyroid carcinoma
PTC: papillary thyroid carcinoma
FNAC: fine-needle aspiration cytology
TI-RADS: thyroid imaging reporting and data system
SVM: support vector machine
ANN: artificial neural network
CNNs: convolutional neural networks
MRF-Net: Multi-Rescale Fusion Network
REI: Rescale Enhancement Image
UNET: Universal Neural Enhancement Technology
ReLU: Rectified Linear Unit
FPN: Feature Pyramid Network
PPV: positive predictive value
NPV: negative predictive value
AUC: area under the curve
DCA: decision curve analysis
TP: true positive
TN: true negative
FP: false positive
FN: false negative
ROC: receiver operating characteristic
AUROC: area under ROC curves
PPV: positive predictive value
NPV: negative predictive value
